# P13 Evaluation of infective endocarditis management in a secondary care hospital: is our service streamlined?

**DOI:** 10.1093/jacamr/dlaf118.020

**Published:** 2025-07-14

**Authors:** Lisa Dwyer-Joyce, Mo Chung Kwok, Stephen Hughes, Nupur Goel

**Affiliations:** Chelsea and Westminster Hospital NHS Foundation Trust, London, UK; Chelsea and Westminster Hospital NHS Foundation Trust, London, UK; Chelsea and Westminster Hospital NHS Foundation Trust, London, UK; Chelsea and Westminster Hospital NHS Foundation Trust, London, UK

## Abstract

**Background:**

Infective endocarditis (IE) is a complex condition which requires multidisciplinary team (MDT) input for effective management. Recommendations for IE management have been outlined by Joint British Societies and The Partial Oral Treatment of Endocarditis (POET) trial. Our 800 bed secondary care trust in London, UK, has one cardiology department providing service across two hospital sites.

**Objectives:**

The aim of this study is to understand our baseline IE service provision and patient outcomes, informing future interventions to enhance local service delivery.

**Methods:**

Patients treated for Gram-positive IE between September 2023 to August 2024, were analysed retrospectively with follow-up data spanning at least six months. Patients were identified using prescription data from the trust’s electronic prescribing system. Adult patients who received at least ten days of IV amoxicillin, flucloxacillin, cefazolin, or vancomycin were screened for inclusion by case note review. Those who received a full course of treatment for IE were included. Data collected included antibiotic regimens (including Outpatient Parenteral Antimicrobial Therapy (OPAT) and oral stepdown), MDT involvement, blood culture results and treatment outcomes. Our service provision was measured against IE guideline recommendations.

**Results:**

The analysis included 31 patients, with a median age of 77 (IQR 53-85), and 74% (*n*=23) were male. IE was associated with prosthetic valve 42% (*n*=13), native valve 42% (*n*=13), and cardiac implantable device 16% (*n*=5). Causative organisms were *Staphylococcus aureus* 35% (*n*=11), *Enterococcus faecalis* 29% (*n*=9), streptococci 19% (*n*=6), coagulase-negative staphylococci 3% (*n*=1), and no identified organism 13% (*n*=4). Echocardiography was performed in 30 patients, with 57% (*n*=17) showing signs of IE. Management decisions involved a specialist IE or cardiothoracic MDT in 39% (*n*=12), separate cardiology plus infection consult 94% (*n*=29), and infection specialist consult only 6% (*n*=2). Additionally, 26% (*n*=8) of patients completed antibiotics via OPAT, and 10% (*n*=3) of patients were switched to oral antibiotics following POET protocol. Incorrect antibiotic treatment occurred for one patient, who was switched to an inappropriate antibiotic without an Infection specialist consult, resulting in a 2 day period of suboptimal therapy. Nine patients had no negative outcomes following treatment. These negative outcomes included all-cause mortality during IE treatment (*n*=4), embolism within 6 months (*n*=3), all-cause rehospitalization for infection (*n*=6) and changes in level of care from pre-admission (*n*=7).

**Conclusions:**

*S. aureus* and *E. faecalis* were the most common organisms identified in this cohort, predominantly involving elderly male patients. These findings align with those observed in other studies, but with increasing incidence of *E. faecalis*. A minority of patients had their antibiotics switched to oral treatment or were managed through OPAT, and one patient received incorrect antibiotic therapy. OPAT and oral step-down strategies need to be utilized to ensure appropriate resource use and reduced length of hospital stay. These results highlight the need for improvements in the trust’s IE service provision, including the formation of a formal local MDT with cardiology, cardiothoracic and infection specialist input, where all IE patients are discussed, with clear documentation of treatment decisions.

